# Analysis of depression incidence and influence factors among middle-aged and elderly diabetic patients in China: based on CHARLS data

**DOI:** 10.1186/s12888-023-05473-6

**Published:** 2024-02-21

**Authors:** Shuo Bai, Jinsong Wang, Jinteng Liu, Yamin Miao, Anqi Zhang, Ziyi Zhang

**Affiliations:** 1 Affiliated Hospital of Yangzhou University, Yangzhou, China; 2 School of Nursing, Yangzhou University, Yangzhou, China

**Keywords:** Middle-aged and elderly, Diabetes mellitus, Depression, Influencing factors, CHARLS

## Abstract

**Background:**

To investigate the incidence of depression in middle-aged and elderly patients with diabetes in China and the influencing factors to provide a theoretical basis to improve the mental health of middle-aged and elderly patients with diabetes and formulate prevention, control, and intervention strategies.

**Methods:**

The sample of this study was obtained from the China Health and Aging Tracking Survey (CHARLS) 2018 survey data, and middle-aged and older patients with diabetes(responding “Yes” to the questionnaire: “Have you ever been told by a doctor that you have diabetes or elevated blood glucose [including abnormal glucose tolerance and elevated fasting glucose]?”) aged ≥ 45 years were selected as study subjects (n = 2,613 ). Depressive symptoms of the study subjects were determined using the simplified version of the Depression Scale for Epidemiological Surveys scores(a score ≥ 10 was defined as depression), influence factors were analyzed using binary logistic regression, and proportion of depressive symptoms was standardized using the sex ratio of the seventh census.

**Results:**

Among the 2,613 middle-aged and elderly patients with diabetes, 1782 (68.2%) had depressive symptoms and 831 (31.8%) had no depressive symptoms. There were 481 (27.0%) patients aged 45−59 years, 978 (54.9%) aged 60−74 years, and 323 (18.1%) aged ≥ 75 years. The depression rate among middle-aged and elderly Chinese patients with diabetes after standardization correction was 67.5%. Binary logistic regression results showed that age, education level, life satisfaction, marital satisfaction, self-rated health grade, somatic pain, visual impairment, physical disability, and the presence of comorbid chronic diseases were factors that influenced the onset of depression in middle-aged and elderly Chinese patients with diabetes (P < 0.05).

**Conclusion:**

According to a survey analysis of the CHARLS 2018 data, depression is influenced by a combination of factors among middle-aged and elderly patients with diabetes in China. Therefore, for this population, targeted prevention and control should be carried out for key populations, such as middle-aged and elderly people, poor physical health, and low life satisfaction and marital satisfaction, from various dimensions (e.g., demographic and sociological factors, physical health status, and life satisfaction and marital satisfaction).

## Background

The prevalence of depression is increasing worldwide, and the increasing aging of the population in China has made depression a major health problem in the elderly. Depression, as one of the most common mental health problems in middle-aged and elderly people, not only has a serious impact on their mental health and quality of life, but is a great burden on families and society [1, 2]. Clinical studies have shown that depression and diabetes often coexist and that people with diabetes are twice as likely to experience depression, increasing the probability of developing diabetes [3].

The latest data released by the International Diabetes Federation in 2019 showed that there are 463 million diabetics worldwide, mainly middle-aged and elderly, and this figure will continue to grow in the future [4]. The psychological comorbidity rate of diabetics is very high due to a number of factors, such as the disease itself and the patients themselves [5]. Depression can significantly reduce the ability of patients with diabetes to self-manage, severely hinder compliance with treatment, making it difficult for caregivers to communicate effectively with patients, and even disrupt the therapeutic relationship; therefore, worsening the condition of diabetics. Some studies have shown that approximately 30% of patients with diabetes have depressive symptoms [6]. Depressed mood has a significant impact on blood glucose levels, the incidence of diabetic complications, cardiovascular events, and even morbidity and mortality in patients with diabetes [7].

Therefore, it is of great practical importance to understand the mental health status of middle-aged and elderly patients with diabetes and to explore as many influencing factors as possible. In this study, we used data from the China Health and Retirement Longitudinal Study (CHARLS) 2018 to analyze the incidence of depression and its influencing factors among middle-aged and elderly patients with diabetes in China, with the aim of providing a reference base to improve the mental health status of middle-aged and elderly patients with diabetes and formulate prevention, control, and intervention strategies. The questionnaire design of this survey refers to international experiences, including the Health and Retirement Survey in the United States and the English Longitudinal Study of Aging. These data are widely recognized and used in academia, allowing for an international comparison of the CHARLS findings with those of other studies. Compared to previous studies, this study has the advantages of broad coverage of the study, large sample size, and representativeness.

## Methods

### Objectives of the study

The sample for this study was obtained from the latest publicly released CHARLS 2018 survey data in September 2020, which covered 28 provinces, 150 districts and counties, and 19,816 respondents in China, with a more authoritative sample representation. The survey’s sampling method uses a multi-stage probability sampling method. The inclusion criteria were the following: (1) age ≥ 45 years; (2) responding “Yes” to the questionnaire “DA007: Have you ever been told by a doctor that you have diabetes or elevated blood glucose (including abnormal glucose tolerance and elevated fasting glucose)?”; (3) provide a clear answer to the Depression Scale (Center for Epidemiologic Studies Depression, CES-D). The exclusion criteria were as follows: (1) lack of information on diabetes; (2) lack of information on depression; and (3) lack of information on relevant covariates. A total sample of 2,613 middle-aged and elderly patients with diabetes were included in the study. This study was approved by the Biomedical Ethics Committee of Peking University (IRB00001052-11015), and informed consent was obtained from all respondents. Basic information on the study participants is presented in Table 1.

**Table 1:** Basic information about the study subjects

Variables	Number	Percentage (%)
Age (years)		
45−59	631	24.15
60−74	1429	54.69
≥ 75	553	21.26
Sex		
Male	1007	38.54
Female	1606	61.46
Place of residence		
Rural	2304	88.17
City	309	11.83
Education level		
Illiterate	1032	39.49
Junior high school and lower	1445	55.30
High school and higher	136	5.20
Religious beliefs		
Yes	314	12.02
No	2299	87.98
Marital Status		
Cohabitation	2320	88.79
Living alone	293	11.21
Smoking		
Yes	635	24.30
No	1978	75.70
Drinking		
Yes	587	22.46
No	2026	77.54
Naps		
Yes	1555	59.51
No	1058	40.49
Life satisfaction		
Satisfaction	2100	80.37
Dissatisfaction	513	19.63
Marital satisfaction		
Satisfaction	2246	85.95
Dissatisfaction	367	14.05
Self-rated health		
Good	252	9.64
General	934	35.74
Not good	667	54.61
Comorbid chronic diseases		
None	1136	43.47
1 kind	810	31.00
2 kinds and above	667	25.53
Somatic pain		
Yes	2040	78.07
No	573	21.93
Hearing impairment		
Yes	836	31.99
No	1777	68.01
Speech disorders		
Yes	59	2.56
No	2554	97.74
Visual impairment		
Yes	705	26.98
No	1908	73.02
Disability		
Yes	524	20.05
No	2089	79.95
Social Interaction		
Yes	1106	42.33
No	1507	57.67
IADL		
Difficulties	6	0.23
No difficulties	2607	99.77

Among the 2,613 middle-aged and elderly patients, 1782 (68.2%) had depressive symptoms, 831 (31.8%) had no depressive symptoms, 640 (35.9%) were males and 1142 (64.1%) were females, 481 (27.0%) were aged 45−59 years, 978 (54.9%) were 60−74 years, and 323 (18.1%) were ≥ 75 years

### Investigation content relevant to this study

Information collected from all study participants includes general information (i.e., age, sex, residence, education, religion, marital status, and social interaction), physical health status (i.e., smoking, alcohol consumption, napping, self-rated health[Would you say your health is good,general or not good?], somatic pain, hearing impairment, speech impairment[Be mute or stutter], visual impairment, disability[physical disabilities or intellectual deficiency], instrumental activities of daily living [IADL], number of comorbid chronic diseases [hypertension, dyslipidemia, malignant tumor, chronic lung disease, heart disease, stroke, arthritis,rheumatism]), marital satisfaction, and life satisfaction.

### Assessment of depressive symptoms

The CHARLS uses a simplified version of the CES-D [8], developed by RADLOFF, which has high reliability and validity in previous studies [9] and can be widely used in middle-aged and older populations. CES-D has 10 items with four scales: “3 = always,” “2 = often,” “1 = sometimes or rarely,” and “0 = never,” where the fifth and eighth items are reverse scored. The scale is rated on a scale of 0−30; the higher the score, the more severe the depression. Based on previous studies of middle-aged and elderly populations, the present study used a critical value of 10 as a classification for the presence or absence of depression, i.e., a score ≥ 10 was defined as depression [8].

### Statistical methods

SPSS software (version 26.0) was used to perform the statistical analysis of the data. The count data were expressed as relative numbers and the χ^2^ test was used to compare between groups. Measurement data with normal distribution were expressed as ± standard deviation and those with non-normal distribution were expressed as M (P25, P75). The analysis of factors influencing depressive symptoms in middle-aged and elderly patients with diabetes was analyzed with binary logistic regression analysis, and the difference was considered statistically significant at P < 0.05. The proportion of depressive symptoms was standardized using census sex ratios.zi.The assignment of independent variables is detailed in Table 2.

**Table 2:** Assignment of independent variables

Variables	Assignment
Age	1 = 45−59; 2 = 60−74; 3 = ≥ 75
Sex	1 = female; 2 = male
Place of residence	1 = city; 2 = rural
Education level	1 = illiterate; 2 = junior high school and lower; 3 = high school and higher
Religious beliefs	1 = no; 2 = yes
Marital status	1 = living alone; 2 = cohabitation
Smoking	1 = no; 2 = yes
Drinking	1 = no; 2 = yes
Naps	1 = no; 2 = yes
Life satisfaction	1 = dissatisfaction; 2 = satisfaction
Marital satisfaction	1 = dissatisfaction; 2 = satisfaction
Self-rated health	1 = not good; 2 = general; 3 = good
Somatic pain	1 = no; 2 = yes
Hearing impairment	1 = no; 2 = yes
Speech disorders	1 = no; 2 = yes
Visual impairment	1 = no; 2 = yes
Disability	1 = no; 2 = yes
Social interaction	1 = no; 2 = yes
IADL	1 = no difficulties; 2 = difficulties
Comorbid chronic diseases	1 = no; 2 = 1 kind; 3 = 2 kinds and more
Depressive	1 = no; 2 = yes

## Results

A univariate analysis of the occurrence of depressive symptoms in middle-aged and elderly patients with diabetes mellitus.

Among the 2,613 middle-aged and elderly patients, 1782 (68.2%) had depressive symptoms, 831 (31.8%) had no depressive symptoms.In people with depression, 640 (35.9%) were males and 1142 (64.1%) were females, 481 (27.0%) were aged 45−59 years old, 978 (54.9%) aged 60−74 years, and 323 (18.1%) aged ≥ 75 years.

There were statistically significant differences (P < 0.05) in age, sex, residence, education, smoking, alcohol consumption, napping, life satisfaction, marital satisfaction, self-rated general health, somatic pain, speech impairment, visual impairment, physical disability, and comorbid chronic diseases between middle-aged and elderly patients with diabetes with and without depressive symptoms (Table 3).

**Table 3:** Comparison of depression levels in elderly diabetic patients with different indicators

Variables	Depression (n = 1782)	No depression (n = 831)	χ^2^	P
Age (years)			43.23	< 0.001
45−59	481 (27.00%)	150 (18.05%)		
60−74	978 (54.88%)	451 (54.27%)		
≥ 75	323 (18.12%)	230 (27.68%)		
Sex			16.28	< 0.001
Male	640 (35.91%)	367 (44.16%)		
Female	1142 (64.09%)	464 (55.84%)		
Place of residence			5.94	0.015
Rural	1590 (89.23%)	714 (85.92%)		
City	192 (10.77%)	117 (14.08%)		
Education level			13.44	0.001
Illiterate	680 (38.16%)	352 (42.36%)		
Junior high school and lower	1023 (57.41%)	422 (50.78)		
High school and higher	79 (4.44%)	57 (6.86%)		
Religious beliefs			2.88	0.09
Yes	201 (11.28%)	113 (13.60%)		
No	1581 (88.72%)	718 (86.40%)		
Marital status			2.95	0.086
Cohabitation	1607 (90.18%)	713 (85.80%)		
Living alone	175 (9.82%)	118 (14.20%)		
Smoking			7.63	0.006
Yes	417 (23.40%)	218 (26.23%)		
No	1375 (76.60%)	603 (73.77%)		
Drinking			5.99	0.014
Yes	376 (21.10%)	211 (25.39%)		
No	1406 (78.90%)	620 (74.61%)		
Naps			7.72	0.005
Yes	1028 (57.69%)	527 (43.42%)		
No	754 (42.31%)	304 (56.58%)		
Life satisfaction			88.88	< 0.001
Satisfaction	1343 (75.36%)	757 (91.10%)		
Dissatisfaction	439 (24.64%)	74 (8.90%)		
Marital satisfaction			57.49	< 0.001
Satisfaction	1469 (82.44%)	777 (93.50%)		
Dissatisfaction	313 (17.56%)	54 (6.50%)		
Self-rated health			155.82	**<** 0.001
Good	102 (5.72%)	150 (18.05%)		
General	576 (32.32%)	358 (43.08%)		
Not good	1102 (61.86%)	323 (38.87%)		
Somatic pain			102.60	**<** 0.001
Yes	1491 (83.67%)	549 (66.06%)		
No	291 (16.33%)	282 (33.94%)		
Hearing impairment			1.34	0.246
Yes	583 (32.72%)	253 (30.45%)		
No	1199 (67.28%)	578 (69.55%)		
Speech disorders			4.82	0.028
Yes	48 (2.69%)	11 (1.32%)		
No	1734 (97.31%)	820 (98.68%)		
Visual impairment			17.50	**<** 0.001
Yes	525 (29.46%)	180 (21.66%)		
No	1257(70.54%)	651 (78.34%)		
Disability			15.60	< 0.001
Yes	395 (22.17%)	129 (15.52%)		
No	1387 (77.83%)	702 (84.48%)		
Social interaction			1.09	0.297
Yes	742 (41.64%)	364 (43.80%)		
No	1040 (58.36%)	467 (56.20%)		
IADL			0.64	0.425
No difficulties	5 (0.28%)	1 (0.12%)		
Difficulties	1777 (99.72%)	830 (99.88%)		
Comorbid chronic diseases			31.30	**<** 0.001
No	718 (40.29%)	418 (50.30%)		
1 kind	558 (31.31%)	252 (30.32%)		
2 kinds and above	506 (28.40%)	161 (19.38%)		

Binary logistic regression analysis of the occurrence of depressive symptoms in middle-aged and elderly patients with diabetes mellitus.

The results of the binary logistic regression analysis showed that age, education level, life satisfaction, marital satisfaction, self-rated health, somatic pain, visual impairment, physical disability, and comorbid chronic diseases were influencing factors (P < 0.05) among middle-aged and elderly patients with diabetes (Table 4).

**Table 4:** Binary logistic regression analysis of depressive symptoms in middle-aged and elderly patients with diabetes

Variables	B	SE	Wald χ^2^	P	OR (95% CI)
Age					
45−59					1
60−74	-0.360	0.124	8.827	0.003	0.698 (0.550, 0.885)
≥ 75	-0.713	0.146	23.800	< 0.001	0.490 (0.368, 0.653)
Sex					
Female					1
Male	-0.237	0.125	3.608	0.058	0.789 (0.618, 1.008)
Education level					
Illiterate					1
Junior high school and lower	0.233	0.105	4.980	0.026	1.263 (1.029, 1.550)
Highschool and higher	-0.096	0.222	0.189	0.664	0.908 (0.588, 1.403)
Place of residence					
City					1
Rural	0.227	0.148	2.345	0.126	1.255 (0.938, 1.677)
Smoking					
No					1
Yes	-0.146	0.131	1.249	0.264	0.864 (0.668, 1.117)
Drinking					
No					1
Yes	0.055	0.118	0.215	0.643	1.056 (0.838, 1.332)
Naps					
No					1
Yes	-0.137	0.096	2.029	0.154	0.872 (0.722, 1.053)
Life satisfaction					
Dissatisfaction					1
Satisfaction	-0.795	0.149	28.577	< 0.001	0.452 (0.337, 0.604)
Marital Satisfaction					
Dissatisfaction					1
Satisfaction	-0.670	0.168	15.859	< 0.001	0.512 (0.368, 0.712)
Self-rated health					
Not good					1
General	-0.488	0.101	23.285	< 0.001	0.614 (0.504, 0.749)
Good	-1.019	0.160	46.598	< 0.001	0.336 (0.246, 0.459)
Somatic pain					
No					1
Yes	0.562	0.111	25.489	< 0.001	1.755 (1.411, 2.183)
Speech disorders					
No					1
Yes	0.513	0.382	1.801	0.180	1.670 (0.790, 3.534)
Visual impairment					
No					1
Yes	0.416	0.111	14.043	< 0.001	1.516 (1.219, 1.884)
Disability					
No					1
Yes	0.321	0.124	6.667	0.010	1.379 (1.080, 1.759)
Comorbid chronic diseases					
No					1
1 kind	0.132	0.108	1.501	0.221	1.142 (0.924, 1.411)
2 kinds and above	0.299	0.123	5.887	0.015	1.349 (1.059, 1.718)

### Standardized correction of the depression rate in middle-aged and elderly diabetic patients

This study normalized the proportion of depressive symptoms using the census sex ratio (female: male = 1:0.95). The adjusted depression rate was 31.0% for males and 36.5% for females, for a total of 67.5% (Table 5).

**Table 5:** Standardized depression rates in middle-aged and elderly Chinese patients with diabetes mellitus

Sex	Standard sex composition ratio	Depression rate	
		Original depression rate	Expected depression rate
Male	48.7%	63.6%	31.0%
Female	51.3%	71.1%	36.5%
Total			67.5%

## Discussion

The results of this study showed that the detection rate of depressive symptoms among middle-aged and elderly patients with diabetes was 67.5%, indicating the severity of mental health problems in this population. In this study, age was an influential factor in the development of depression in middle-aged and elderly patients with diabetes. The results also showed that the prevalence of depression was lower in the age group 60−74 years and ≥ 75 years than in the age group 45−59 years; the younger the age, the higher the prevalence of depression. The results of this study are consistent with the findings of Majumdar et al. [10], which can be explained by the fact that the age group 45−59 years has higher expectations of life and faces great stress in both life and work. A study in South Asia has confirmed that family status, financial problems, physical illness, and sudden accidents contribute to the increased risk of depression in the age group 45−59 years [11]. Meanwhile, participants aged 45−59 years are in the middle stage of Chinese society and have to face the double responsibility of taking care of their children and parents, which is stressful; therefore, the prevalence of depression is higher in this age group. The present study showed that education level of middle school or lower was a risk factor for depression compared to illiterate middle-aged and elderly patients with diabetes. However, some studies [12, 13] found that low education level was a risk factor for the occurrence of depression in patients with diabetes, which is not consistent with the results of the present study, and specific reasons need to be studied further.

Self-rated health (SRH) is an individual’s perception of their health and has been widely recommended by the World Health Organization for personal investigation of their level of health [14]. SRH has also been suggested to be a predictor of the occurrence of diseases such as cardiovascular events [15, 16] and mental disorders [17]. In this study, SRH was an influential factor in the occurrence of depression in middle-aged and elderly diabetic patients. Ghislaine et al. [18] found that self-assessed health status was a significant predictor of the onset of major depression in patients with type II diabetes through a follow-up survey in 2013. Furthermore, Huang et al. [17] found that older adults with poorer SRH status had a higher risk of depression, both of which are consistent with the results of this study. Therefore, healthcare workers, as well as community workers, should pay attention to the assessment of health status in middle-aged and elderly patients with diabetes. In addition, this study showed that the combination of multiple chronic diseases was an influential factor in the development of depression in middle-aged and elderly diabetic patients. A meta-analysis study by Huang et al. [19] found that heart disease, stroke, obstructive pulmonary disease, and diabetes were risk factors for depression in the elderly. A survey by Patten et al. [20] showed that the incidence of depression in the elderly was significantly associated with chronic diseases characterized by inflammation and pain. Diabetes itself is a type of chronic disease that already interferes with the daily life of patients, and this effect is undoubtedly more severe when middle-aged and elderly people suffer from other chronic diseases, increasing the risk of depression in middle-aged and elderly patients with diabetes. In addition, using CHARLS2011 baseline data and 2013 follow-up data, some studies found that depression was more prevalent among elderly patients with chronic diseases and that the number of chronic disease comorbidities was positively associated with the risk of depression [21], which confirms the results of this study.

Diabetes is accompanied by various complications, such as neuropathy, diabetic foot, and retinopathy [22], which can cause physical disability, pain, and vision loss, and place a great burden on middle-aged and elderly patients and their families. The results of this study showed that middle-aged and elderly diabetic patients with physical pain had a higher detection rate of depressive symptoms than those without physical pain. Neuropathy becomes a common complication in patients with long-term diabetes, which can lead to pain, sensory loss, and even amputation [23], and this neuropathic pain affects one-third of diabetic patients [24]. A meta-analysis that included low- and middle-income countries and involved nearly 300,000 people showed that pain and depression were highly correlated [25]. Meanwhile, a study in the United States [26] showed that individuals with long-term physical disabilities suffer higher levels of pain and pain interference compared to normal individuals. Furthermore, Mathew et al. [27] identified physical disability as a risk factor for depression in patients with diabetes through a cross-sectional survey. This further confirms why middle-aged and older patients with diabetes with physical disabilities are at increased risk of depression. Middle-aged and elderly people with diabetes are a special group in their own right, and with pain and physical disability, their health status is worse and they are more likely to suffer from mental health problems. Diabetic retinopathy (DR), a major retinal vascular complication of diabetes, is a progressive disease and one of the leading causes of visual impairment and blindness in diabetic patients [28], and the results of this study showed that middle-aged and older diabetic patients with visual impairment had a higher incidence of depression. Dornan et al. [29] found that older patients with diabetes with visual impairment had a much poorer health status and were more frequently depressed than those without visual impairment. The results of a population-based systematic review by Gianni et al. [30] showed that visual impairment was significantly associated with the occurrence of depression. Older adults with visual impairment have a reduced ability to perform daily activities, and their probability of falling and social isolation is higher [31], therefore, in conjunction with the disease, middle-aged and older patients with diabetes with visual impairment are more likely to experience depression. Therefore, more care and assistance should be provided to middle-aged and elderly patients with diabetes with somatic pain, physical disability, and visual impairment.

This study also found that middle-aged and older patients with diabetes who were satisfied with their lives had a lower risk of depression compared to those who were dissatisfied with their lives and that middle-aged and older patients with diabetes who were satisfied with their marriage had a lower risk of developing depressive symptoms compared to those who were dissatisfied with their marriages. A previous study [32] showed that depressive symptoms were significantly associated with life satisfaction in middle-aged and elderly Chinese patients with diabetes, and when middle-aged and elderly people have diabetes, their dissatisfaction with life increases greatly due to decreased quality of life, indirectly increasing their risk of depression. Min et al. [33] found that spousal care and marital satisfaction were important moderators of the onset of depression in patients with chronic disease. Furthermore, a recent study [34] extended the study from the individual to the couple level using marriage as an important social context, and this binary approach study confirmed that one’s health has an impact on both the depressive symptoms of the individual and the partner, which implies that high marital satisfaction represents a high quality couple relationship and that a high quality couple relationship can help both partners mitigate the effects and negative experiences of negative events in their lives, and, conversely, it can exacerbate the negative experiences of both partners and therefore increase the risk of depression.

All the data in this study came from the CHARLS database. The standard of inclusion of diabetic patients was oral questioning without clinical diagnosis, which may lead to bias of case data, which is the limitation of this study.In addition,most of the sample in the study was rural, female and less educated. These factors may have contributed to the higher prevalence of depression in this study.

## Conclusion

In this study, the incidence of depression in middle-aged and elderly diabetic patients in China is high and is affected by multiple factors. To improve the mental health of middle-aged and elderly diabetic patients in China and allow them to lead a more active and healthy life, it is recommended that communities and hospitals increase caregiving services for middle-aged and elderly diabetic patients, strengthen the training of relevant professional caregivers, and focus on middle-aged and elderly diabetic patients aged 45−59 years with poor self-assessed health status, multiple comorbid chronic diseases, somatic pain, physical disability, visual impairment, and low satisfaction with life and marriage. In addition, the factors included in this study were based on the CHARLS database and the variables included were limited. The specific conditions of patients with diabetes in the sample could not be classified, and the results obtained may have some limitations.

## Data Availability

The datasets used and analyzed in the current study are available upon reasonable request from the official CHARLS application.
